# Supplementary material for: Hotspots of aberrant enhancer activity punctuate the colorectal cancer epigenome
Source: Nat Commun. 2017 Feb 7;8:14400. doi: 10.1038/ncomms14400 (PMC5309719; doi:10.1038/ncomms14400)
Supplement: Supplementary Information — Supplementary Figures, Supplementary Table. [file ncomms14400-s10.pdf]

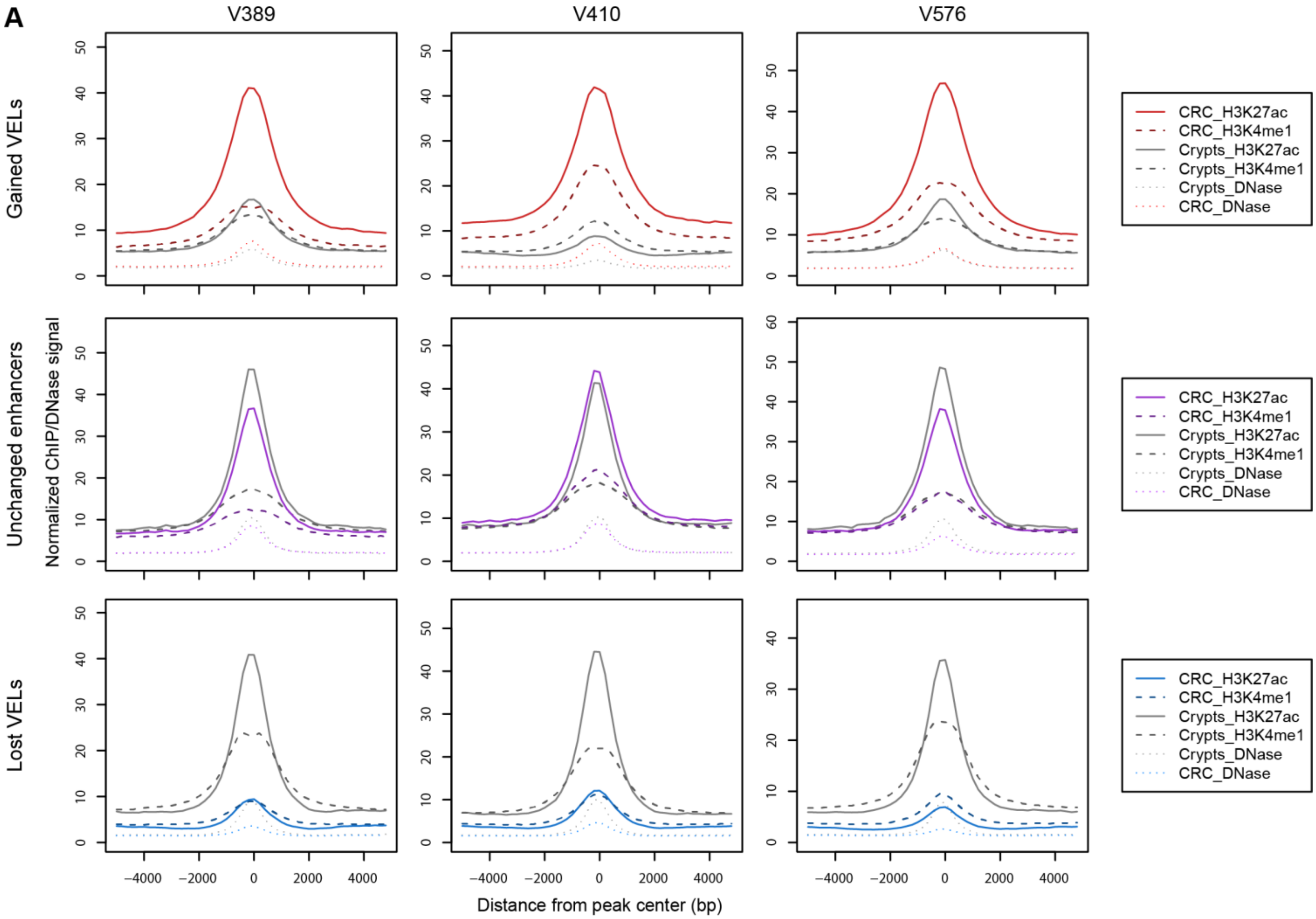

A
V389
V410
V576
Gained VELs
Unchanged enhancers
Lost VELs
Normalized ChIP/DNase signal
Distance from peak center (bp)
CRC_H3K27ac
CRC_H3K4me1
Crypts_H3K27ac
Crypts_H3K4me1
Crypts_DNase
CRC_DNase

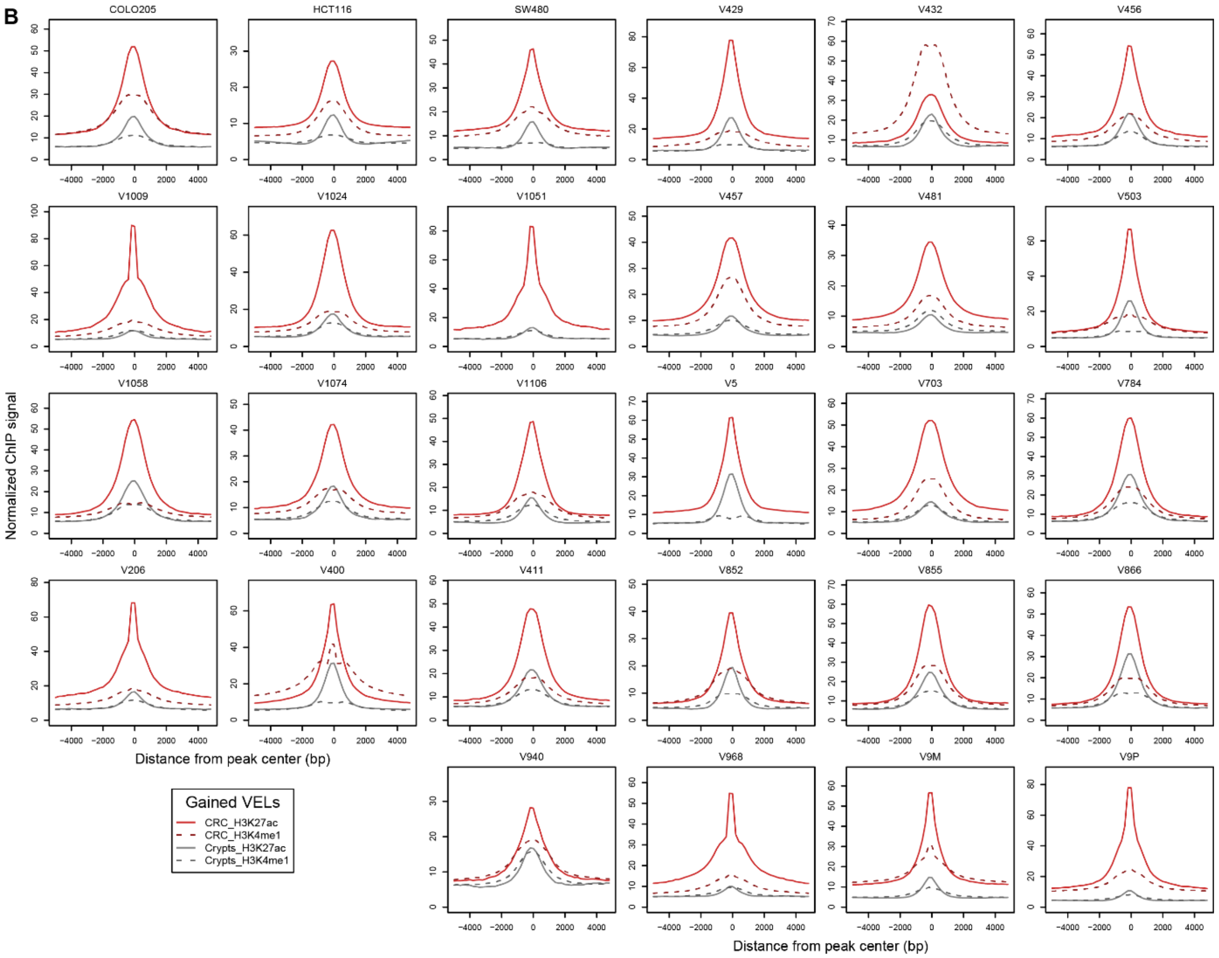
B
COLOZ05
HCT116
SW480
V429
V432
V456
V1009
V1024
V1051
V457
V481
V503
V1058
V1074
V1106
V5
V703
V784
V206
V400
V411
V852
V855
V866
V940
V968
V9M
V9P
Normalized ChIP signal
Distance from peak center (bp)
Gained VELs
CRC_H3K27ac
CRC_H3K4me1
Crypts_H3K27ac
Crypts_H3K4me1

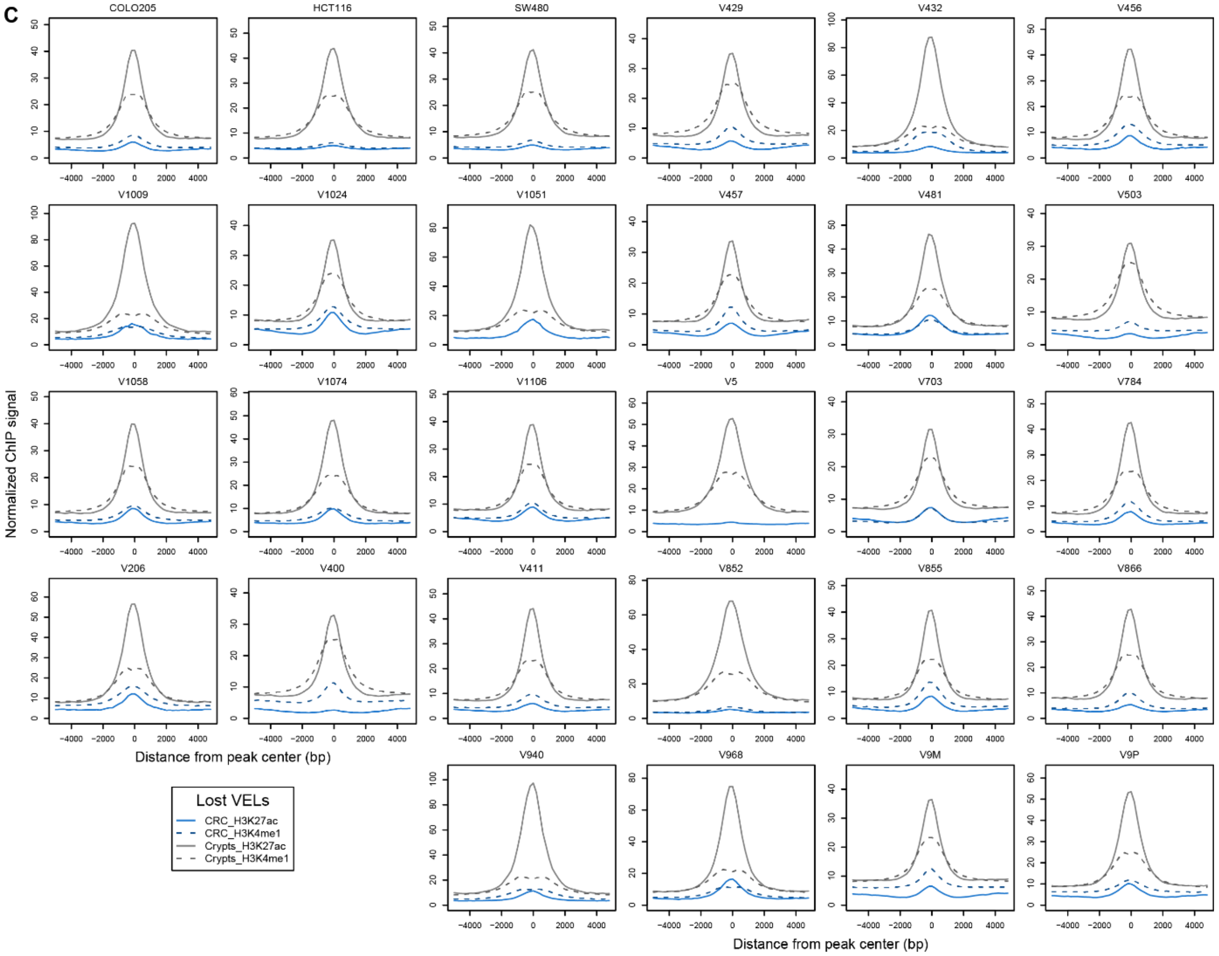
C
COLO205
HCT116
SW480
V429
V432
V456
V1009
V1024
V1051
V457
V481
V503
V1058
V1074
V1106
V5
V703
V784
V206
V400
V411
V852
V855
V866
V940
V968
V9M
V9P
Normalized ChIP signal
Distance from peak center (bp)
Lost VELs
CRC_H3K27ac
CRC_H3K4me1
Crypts_H3K27ac
Crypts_H3K4me1

D

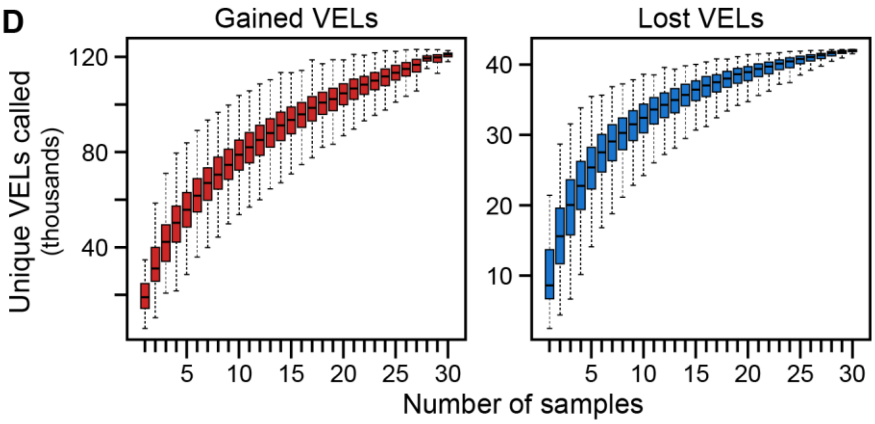

Supplementary Fig 1 –

A-C) Aggregate plots show average of normalized H3K27ac (solid lines) and H3K4me1 (dashed lines) ChIP-seq and DHS (dotted lines) enrichment in normal colon crypt (gray) and each CRC cell line (color), for (A) three CRC cell lines with DHS profiles and (B) and (C) 28 CRC lines without.

D) Saturation plots displaying the number of unique gained (left) and lost (right) VELs identified as a function of the number of CRC samples profiled. The line within the box represents the median, upper and lower box boundaries represent the 1st and 3rd quartiles, and whiskers extend to the extremes of the data.

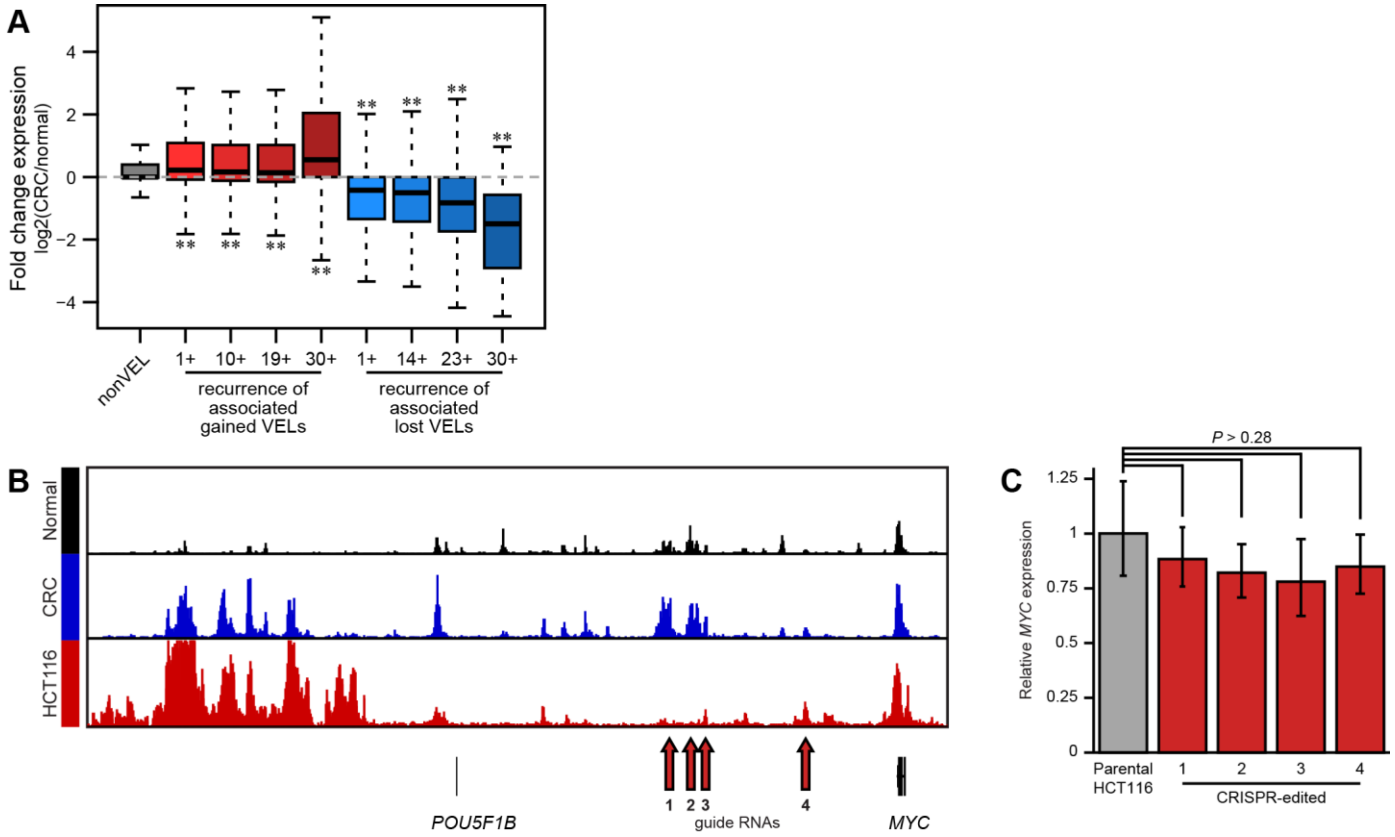

Supplementary Fig 2 –

A) Fold change of expression of genes associated with gained (red) and lost (blue) VELs of various recurrence levels, and genes not associated with VELs (nonVEL; gray) across all CRC cell lines. **MWW P < 1×10^{-31} versus non-VEL genes. The line within the box represents the median, upper and lower box boundaries represent the 1st and 3rd quartiles, and whiskers extend to the nearer of the extremes of the data or 1.5 times the interquartile range with outliers suppressed.

B) Normalized H3K27ac ChIP-seq track for HCT116 compared to CRC and normal colon H3K27ac super-tracks (median binned signal of all normal crypts or all CRC cell lines) at the *MYC* locus. Guide RNA target sites for the four CRISPR-Cas9 edits performed are indicated by arrows.

C) Barplots indicate *MYC* RNA levels in the parental HCT116 cell line and cells disrupted by CRISPR/Cas9 at each of the loci indicated in B (relative to GAPDH, mean ± 95% confidence interval of quadruplicates).

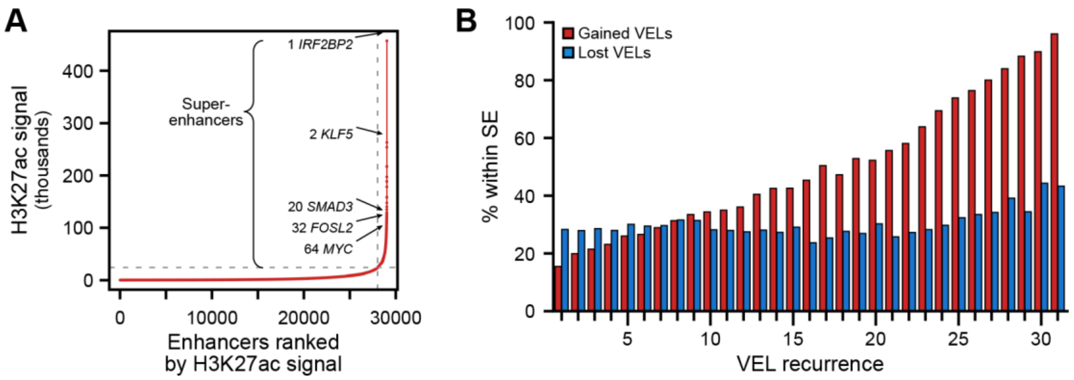

Supplementary Fig 3 –

A) Ranked plot of H3K27ac ChIP-seq signals in a representative CRC cell line (V410). Points above and right of grey dashed lines correspond to super enhancers (SE) as determined using the ROSE algorithm.

B) Percentage of VELs within super enhancers (SE) as a function of VEL recurrence.

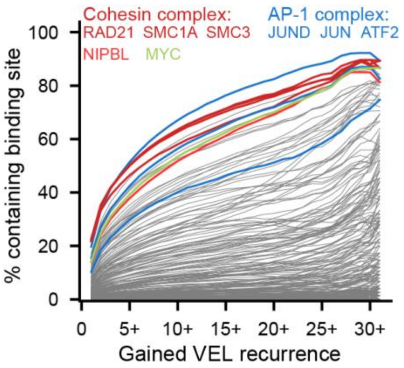

Supplementary Fig 4 –
Percentage of gained VELs bound by a given factor as a function of VEL recurrence.

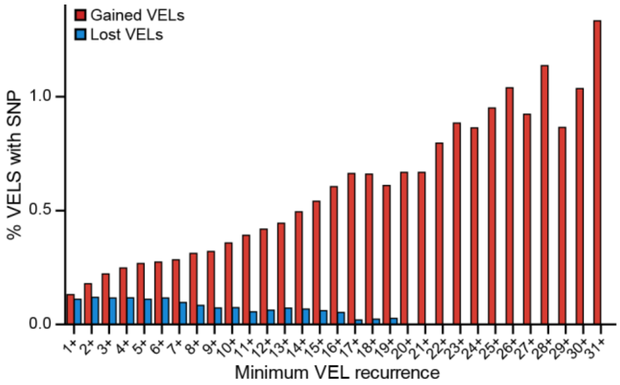

Supplementary Fig 5 –
Percentage of gained (red) and lost (blue) VELs that contain a risk SNP (lead or LD) as a function of VEL recurrence.

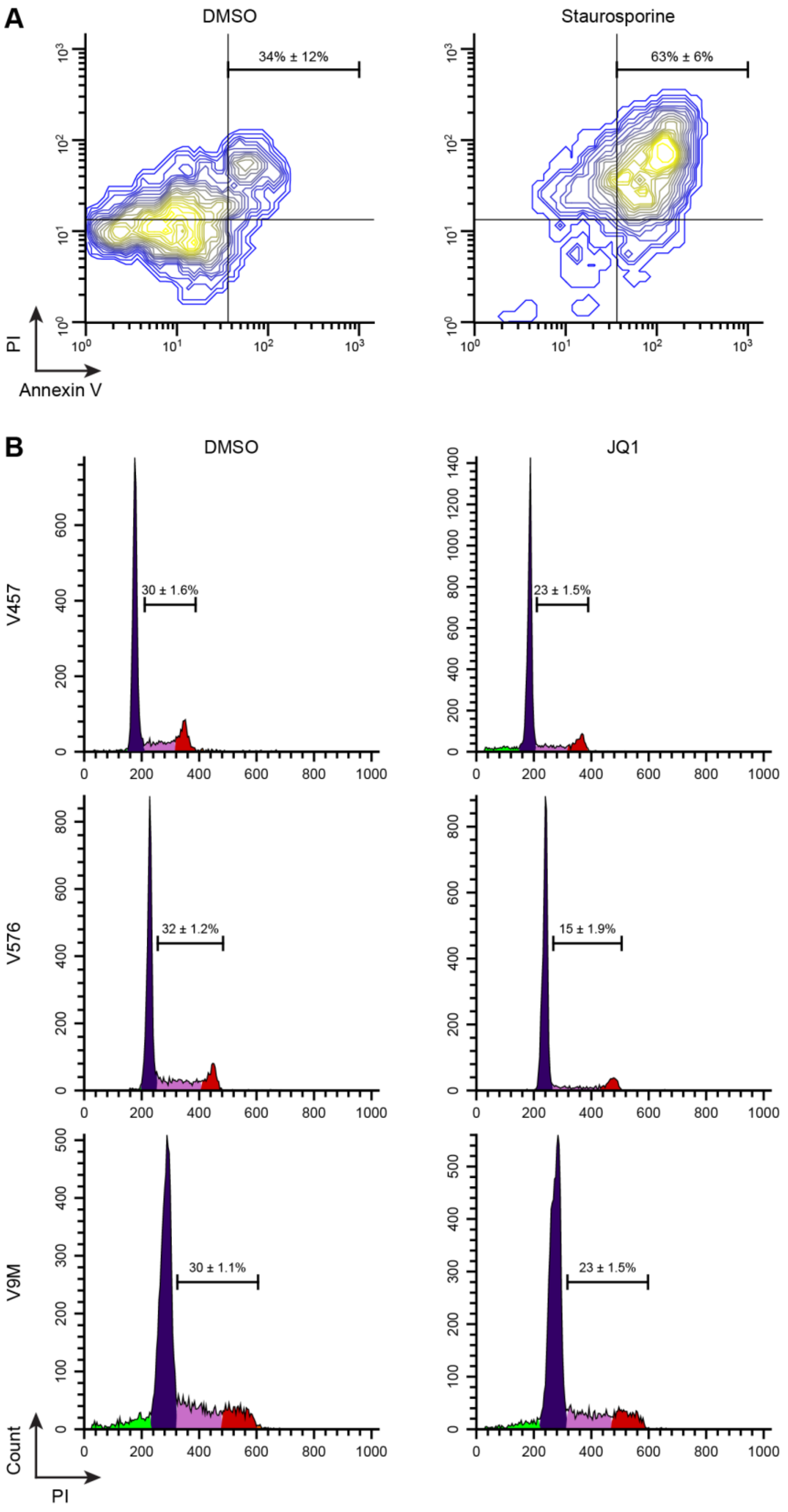

Supplementary Fig 6 –

A) Apoptosis analysis by flow cytometry of Annexin-V and propidium iodide (PI) stained cells for JQ1 treated (right), DMSO (negative control; left) or staurosporine (positive control; center) cells in a relatively sensitive CRC line (V457). Percentages of apoptotic cells are shown (mean ± standard deviation of duplicates).

B) Cell cycle analysis by flow cytometry of propidium iodide (PI) stained cells for JQ1 treated (right) or DMSO (control; left) cells in three CRC lines. Percentages of replicating (S and G2/M) cells are shown (mean ± standard deviation of duplicates).

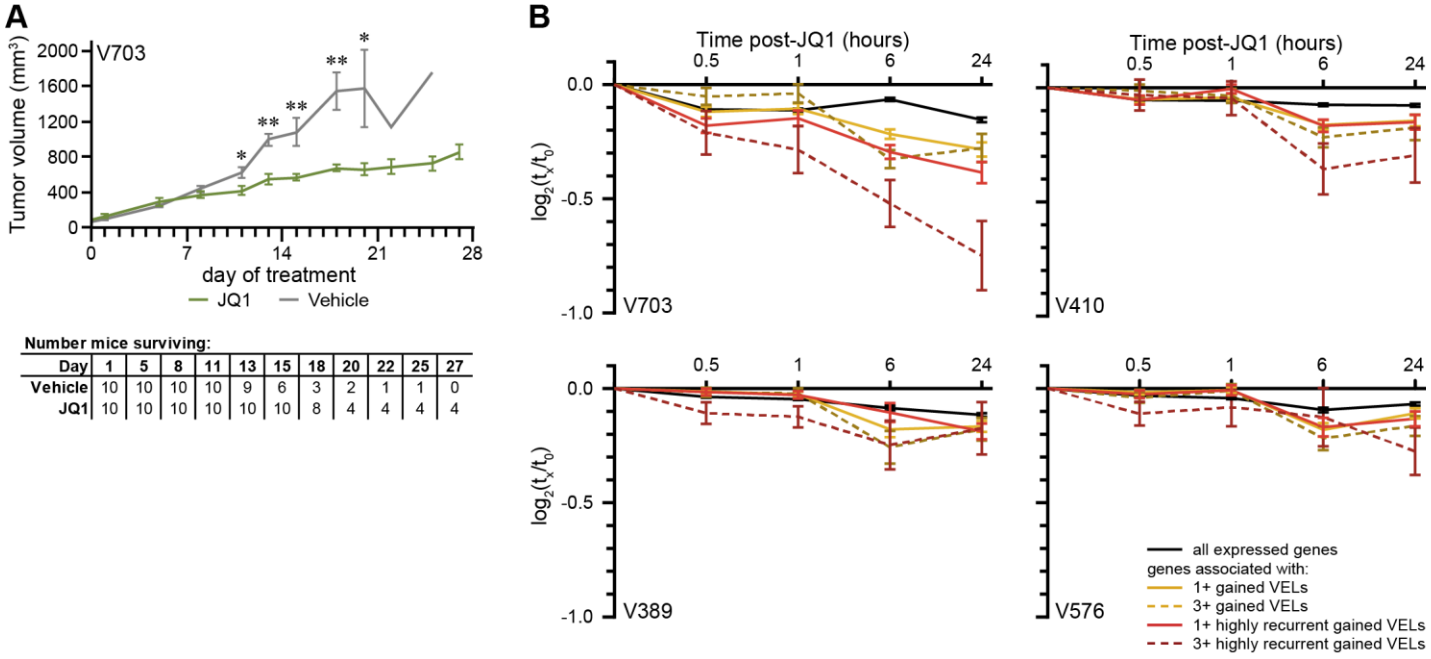

Number mice surviving:

Day	1	5	8	11	13	15	18	20	22	25	27
Vehicle	10	10	10	10	9	6	3	2	1	1	0
JQ1	10	10	10	10	10	10	8	4	4	4	4

Supplementary Fig 7 –

A) Tumor volume in mouse xenograft models treated with 25 mg/kg BID JQ1 (green) or DMSO control (grey) in a relatively sensitive CRC line (V703; mean ± standard deviation). T-test *P < 0.05, **P < 0.01; n = 10 per group. Table below indicates the number of remaining mice in each cohort at each day tumor measurements were taken.

B) Line plots depicting fold change of expression (JQ1 treated/untreated) of various gene sets at indicated time points of JQ1 treatment (mean ± SEM).

Supplementary Table 1: Overview of ChIP-seq and DNase-seq samples

sample	sample type	Tumor stage	H3K27ac	H3K4me1	H3K27me3	DNase
C28	normal	N/A	X	X	X	
C29	normal	N/A	X	X		
C31	normal	N/A				X
C34	normal	N/A				X
C36	normal	N/A				X
C37	normal	N/A	X	X		
Crypt1	normal	N/A	X			
Crypt2	normal	N/A	X			
Crypt3	normal	N/A	X			
Crypt5	normal	N/A	X	X		
COLO205	CRC cell line	D	X	X		
HCT116	CRC cell line	unknown	X	X		
SW480	CRC cell line	B	X	X		
V1009	CRC cell line	B	X	X		
V1024	CRC cell line	B	X	X		
V1051	CRC cell line	B	X			
V1058	CRC cell line	B	X	X		
V1074	CRC cell line	B	X	X		
V1106	CRC cell line	B	X	X		
V206	CRC cell line	B	X	X		
V389	CRC cell line	C	X	X		X
V400	CRC cell line	liver metastasis	X	X		
V410	CRC cell line	liver metastasis	X	X	X	X
V411	CRC cell line	adenoma*	X	X		
V429	CRC cell line	B	X	X		
V432	CRC cell line	B	X	X		
V456	CRC cell line	B	X	X		
V457	CRC cell line	liver metastasis	X	X		
V481	CRC cell line	B	X	X		
V5	CRC cell line	C	X			
V503	CRC cell line	liver metastasis	X	X		
V576	CRC cell line	liver metastasis	X	X		X
V703	CRC cell line	B	X	X		
V784	CRC cell line	liver metastasis	X	X		
V8	CRC cell line	D		X		
V852	CRC cell line	D	X	X		
V855	CRC cell line	liver metastasis	X	X		
V866	CRC cell line	liver metastasis	X	X		
V868	CRC cell line	liver metastasis		X		
V940	CRC cell line	B	X	X		
V968	CRC cell line	B	X	X		
V9M	CRC cell line	liver metastasis	X	X		
V9P	CRC cell line	D	X	X		
CRC_17A	primary CRC	unknown	X	X		
CRC_23A	primary CRC	unknown	X	X		
CRC_6A	primary CRC	unknown	X	X		
CRC_7A	primary CRC	unknown	X	X		
FAP5_polyp1	adenoma	adenoma	X			
FAP5_polyp2	adenoma	adenoma	X			
**TOTAL**			**44**	**39**	**2**	**6**

*V411 was derived from a xenograft of a colon adenoma cell line
